# Review of Equity of Access to Treatment for Gambling Harms in Racial and Ethnic Minority Populations: A Mixed Methods Study

**DOI:** 10.1192/bjo.2024.466

**Published:** 2024-08-01

**Authors:** Deborah Davidson-Hine, Helen Lloyd, James Close, Konstantinos Ioannidis, Mat King

**Affiliations:** 1 Southern Health Foundation Trust - Southern Gambling Service, Southampton, United Kingdom; 2 School of Psychology, University of Plymouth, Plymouth, United Kingdom

## Abstract

**Aims:**

The NHS Southern Gambling Service (SGS) is a service providing evidence-based assessment and treatment for people affected by Gambling Disorder (GD) across the South-East of England. This service evaluation aimed to ascertain whether SGS was offering equality of access to treatment and suitable provision of treatment to ethnic minority communities, and whether there were barriers making it difficult for people from ethnic minority communities to access and engage in treatment for gambling harms.

**Methods:**

Quantitative ethnic origin demographic data was obtained from 120 referrals to SGS between September 2022 and October 2023. These were statistically compared with the ethnic origin demographics of the general population in the same geographical area, as identified by Office of National Statistics (ONS) Census 2021 data. Qualitative data was collected through interviews with three participants from ethnic minority populations who were engaged in treatment with the service. Relevant themes in the qualitative data were identified using thematic analysis.

**Results:**

Quantitative data results indicated no significant statistical differences in most ethnic origin categories between the proportion of referrals to SGS from the ethnic origin and the recorded proportion of this ethnic origin in the general population. There was a greater difference for the “other ethnic group” category (chi square p,0.05, uncorrected), which was likely due to a difference in categorisation of ethnicity between SGS and ONS in 2021 Census.

The qualitative review identified themes of value of money, stigmatisation, different cultural attitudes towards gambling, and experiences of healthcare. GPs were identified as the first step towards seeking help for gambling.

**Conclusion:**

These results suggested that SGS was offering equality of access to treatment for people from ethnic minority populations and that there were not significant barriers preventing people from ethnic minority populations accessing treatment. The reported positive experiences of participants' referral to and treatment with SGS indicates that for these participants suitable provision of treatment had been offered by our service.

With the thematic analysis identifying GPs as the first step towards seeking help for their gambling, this study indicates the importance of the gambling service working closely with primary care for the equitable access to treatment from gambling harms on a regional level.

These preliminary findings are based on a limited, small sample. Further research using a larger, more diverse sample to gain a deeper knowledge would be advised to further shape the service offer to ensure equity of access.

